# Time-Adaptive Statistical Test for Random Number Generators

**DOI:** 10.3390/e22060630

**Published:** 2020-06-07

**Authors:** Boris Ryabko

**Affiliations:** 1 Institute of Computational Technologies of the Siberian Branch of the Russian Academy of Science, 630090 Novosibirsk, Russia; boris@ryabko.net; 2 Department of Information Technologies, Novosibirsk State University, 630090 Novosibirsk, Russia

**Keywords:** hypothesis testing, randomness testing, random number generators, test battery, *p*-value

## Abstract

The problem of constructing effective statistical tests for random number generators (RNG) is considered. Currently, there are hundreds of RNG statistical tests that are often combined into so-called batteries, each containing from a dozen to more than one hundred tests. When a battery test is used, it is applied to a sequence generated by the RNG, and the calculation time is determined by the length of the sequence and the number of tests. Generally speaking, the longer is the sequence, the smaller are the deviations from randomness that can be found by a specific test. Thus, when a battery is applied, on the one hand, the “better” are the tests in the battery, the more chances there are to reject a “bad” RNG. On the other hand, the larger is the battery, the less time it can spend on each test and, therefore, the shorter is the test sequence. In turn, this reduces the ability to find small deviations from randomness. To reduce this trade-off, we propose an adaptive way to use batteries (and other sets) of tests, which requires less time but, in a certain sense, preserves the power of the original battery. We call this method time-adaptive battery of tests. The suggested method is based on the theorem which describes asymptotic properties of the so-called *p*-values of tests. Namely, the theorem claims that, if the RNG can be modeled by a stationary ergodic source, the value $-log\;\pi (x_{1}x_{2}\ldots x_{n})/n$ goes to 1−h when *n* grows, where $x_{1}x_{2}\ldots$ is the sequence, π() is the *p*-value of the most powerful test, and *h* is the limit Shannon entropy of the source.

## 1. Introduction

Randomness has many applications in cryptography, statistical sampling, computer modeling, and numerical Monte Carlo methods, as well as in games, gambling, and other fields. In practice, random numbers are created by devices that generate a sequence of numbers or characters. These devices are called random number generators (RNGs) and pseudo random number generators (PRNGs). RNGs are based on physical sources, while pseudo random numbers are generated by computer programs. The goal of RNG and PRNG is to generate sequences of binary digits that are distributed as a result of throwing an “honest” coin or, more precisely, obey the Bernoulli distribution with parameters (1/2,1/2). As a rule, for practically used RNGs and PRNGs, this property is verified experimentally using statistical tests developed for this purpose. Currently, there are more than one hundred applicable statistical tests, as well as dozens RNGs based on different physical processes, and an even greater number of PRNGs based on different mathematical algorithms (see for review [1,2,3]).

Informally, an ideal RNG should generate sequences that pass all tests. In practice, especially in cryptographic applications, this requirement is formulated as follows: an RNG must pass a so-called battery of statistical tests, that is, some fixed set of tests. When a battery is applied, each test in the test battery is applied separately to the RNG. Among these batteries, we mention the Marsaglia’s Diehard battery, which contains 16 tests [4], the National Institute of Standards and Technology (NIST) battery of 15 tests [5], several batteries proposed by L’Ecuyer and Simard [2], which contain from 10 to 106 tests, and many others (see for review [1,2,6]). In addition, these batteries contain many tests that can be used with different values of the parameters, potentially increasing the total number of tests in the battery. Note that practically used RNG should be tested from time to time as with any physical equipment, and therefore these test batteries should be used continuously.

How show large batteries of tests be evaluated? On the one hand, the larger is the test battery, the more likely it is to find flaws in the tested RNG. On the other hand, the larger is the battery, the more time is required for testing. (Thus, L’Ecuyer and Simard [2] remarked the need for small batteries to increase computational efficiency.) Another view is as follows: in reality, the time available to study any RNG is limited. Given a certain time budget, one can either use more tests and relatively short sequences generated by the RNG, or use fewer tests, but longer sequences and, in turn, this gives more chances to find deviations of randomness of the considered RNG.

To reduce this trade-off, we propose a time-adaptive testing of RNGs, in which, informally speaking, first all the tests are executed on relatively short sequences generated by the RNG, and then a few “promising” tests are applied for the final testing. Of course, the key question here is which tests are promising. For example, if a battery of two tests is applied to (relatively short) sequences of the same length, it can be assumed that the smaller is the *p*-value, the more promising is the test. However, a more complicated situation may arise when we have to compare two tests that were applied to sequences of different lengths (for example, the first test was applied to a sequence of length $l_{1}$, and the second to a sequence of length of $l_{2}$, $l_{1}\neq l_{2}$). We show that, if our goal is to choose the most powerful test, then a good strategy is to choose the test *i* for which the ratio $-\log\left(p_{-value_{i}}\right)/l_{i}$ is maximum. This recommendation is based on the following theorem: if an RNG can be modeled by a stationary ergodic source, the value $-log\;\pi (x_{1}x_{2}\ldots x_{n})/n$ goes to 1−h, if *n* grows, where $x_{1}x_{2}\ldots$ is a generated sequence, π() is the *p*-value of the most powerful test, and *h* is the limit Shannon entropy of the stationary ergodic source μ. (Here, the Shannon entropy of order *m*, m=1,2,…, is defined by $h_{m}=$$-\sum_{u\in {\left\{0,1\right\}}^{m-1}}$$\mu \left(u\right)\sum_{v\in \{0,1\}}$μ(v/u)logμ(v/u) and the limit Shannon entropy is defined by $h=\lim_{m\to \infty }h_{m};\;$ see [7].) This theorem plays an important rule in the suggested time-adaptive scheme and is described in the first part of the paper, whereas the time-adaptive testing is described afterwards. The description is illustrated by experiments with the battery Rabbit from [2].

As far as we know, the proposed approach to testing RNGs is new, but the idea of finding the best test among many, testing the tests step by step in an increasing sequence, is widely used in algorithmic information theory, where the notion of random sequence is formally investigated and discussed [8,9,10].

## 2. Hypothesis Testing and Properties of *P*-Values

### 2.1. Notation

We consider RNG which generates a sequence of letters $x=x_{1}x_{2}$$\ldots x_{n},$n≥1, from a finite alphabet ${\left\{0,1\right\}}^{n}$. Two statistical hypotheses are considered: $H_{0}=$ {x *obeys the uniform distribution ($\mu_{U}$) on ${\left\{0,1\right\}}^{n}\}$*, and the alternative hypothesis $H_{1}={\bar{H}}_{0}$, that is, $H_{1}$ is the negation of $H_{0}$. It is a particular case of the so-called goodness-of-fit problem, and any test for it is called a test of fit, see [11]. Let *t* be a test. Then, by definition, the significance level α equals the probability of the Type I error, α∈(0,1). Denote a critical region of the test *t* for the significance level α by $C_{t}\left(\alpha \right)$ and let ${\bar{C}}_{t}\left(\alpha \right)$$={\left\{0,1\right\}}^{n}\setminus C_{t}\left(\alpha \right).$ (Recall that Type I error occurs if $H_{0}$ is true and is rejected. Type II error occurs if $H_{1}$ is true, but $H_{0}$ is accepted. Besides, for a certain $x=x_{1}x_{2}\ldots x_{n}\;\;$ $H_{0}$ is rejected if and only if $x\in C_{t}\left(\alpha \right)$.)

Suppose that $H_{1}$ is true, and the investigated sequence $x=x_{1}x_{2}\ldots x_{n}$ is generated by an (unknown) source ν. By definition, a test *t* is consistent, if, for any significance level α∈(0,1), the probability of Type II error goes to 0, that is

(1) $$\lim_{n\to \infty }\nu \left({\bar{C}}_{t}\left(\alpha \right)\right)=0\;.$$

Suppose that $H_{1}$ is true and the sequences $x\in {\left\{0,1\right\}}^{n}$ obey a certain distribution ν. It is well-known in mathematical statistics that the optimal test (Neyman–Pearson or NP test) is described by the Neyman–Pearson lemma and the critical region of this test is defined as follows:

$$C_{NP}\left(\alpha \right)=\left\{x:\;\;\mu_{U}\left(x\right)/\nu \left(x\right)\leq \lambda_{\alpha }\right\}\;,$$

where α∈(0,1) is the significance level and the constant $\lambda_{\alpha }$ is chosen in such a way that $\mu_{U}\left(C_{NP}\left(\alpha \right)\right)=\alpha$ (see [11]). (We did not take into account that the set ${\left\{0,1\right\}}^{n}$ is finite. Strictly speaking, in such a case, a randomized test should be used, but in what follows we consider asymptotic behavior of tests for large *n*, and this effect would be negligible). Note that, by definition, $\mu_{U}\left(x\right)=2^{-n}$ for any *x*$\in {\left\{0,1\right\}}^{n}$.

### 2.2. The P-Value and Its Properties

The notion of the critical region is connected with the so-called *p*-value, which we define for the NP-test by the following equation:

(2) $$\pi_{NP}\left(x\right)=\mu_{U}\left\{y:\nu \left(y\right)>\nu \left(x\right)\right\}=\left|\left\{y:\nu \left(y\right)>\nu \left(x\right)\right\}\right|/2^{n}\;.$$

Informally, $\pi_{NP}\left(x\right)$ is the probability to meet a random point *y* which is “worse” than the observed when considering the null hypothesis.

The NP-test is optimal in the sense that its probability of a Type II error is minimal, but when testing an RNG the alternative distribution is unknown, and, hence, different tests are necessary. Let us consider a certain statistic τ (that is, a function on ${\left\{0,1\right\}}^{n}$), and define the *p*-value for this τ and *x* as follows:

(3) $$\pi_{\tau }\left(x\right)=\mu_{U}\left\{y:\tau \left(y\right)>\tau \left(x\right)\right\}=\left|\left\{y:\tau \left(y\right)>\tau \left(x\right)\right\}\right|/2^{n}\;.$$

(Note that the definition $\pi_{NP}$ in Equation (2) corresponds to this equation if the value ν(x) is considered as a statistic, i.e., τ(x)=ν(x)).

### 2.3. The P-Value and Shannon Entropy

It turns out that there exist such tests whose asymptotic behavior is close to that of the NP-test for any (unknown) stationary ergodic source ν (see [12]). Those tests are based on so-called universal codes (or data-compressors) and are described in [13,14], where it is shown that they are consistent. We describe those tests in Appendix A and show that they are asymptotically optimal. The following theorem describes the asymptotic behavior of *p*-values for stationary ergodic sources for NP test and the above-mentioned tests, which are based on universal codes (see Appendix A). We use this theorem as the theoretical basis for adaptive statistical testing developed in this paper.

**Theorem 1.**  *(i) If ν is a stationary ergodic measure, then, with probability 1,*

(4) $$\lim_{n\to \infty }-\frac{1}{n}\log\pi_{NP}\left(x\right)=1-h\left(\nu \right)\;,$$

*where h(ν) is the Shannon entropy of ν (see for definition [7]).*

*(ii) There exists such a statistic τ that, for any stationary ergodic measure ν, with probability 1,*

(5) $$\lim_{n\to \infty }-\frac{1}{n}\log\pi_{\tau }\left(x\right)=1-h\left(\nu \right)\;,$$

*where p-values $\pi_{NP}$ and $\pi_{\tau }$ are defined in Equations (2) and (3), correspondingly.*

The statistic τ and the corresponding test of fit are described in Appendix A and the proof of the theorem is given in Appendix B, but here we note that this theorem gives some idea of the relation between the Shannon entropy of the (unknown) process ν and the required sample size. Indeed, suppose that the NP test is used and the desired significance level is α. Then, we can see that (asymptotically) α should be larger than $\pi_{NP}\left(x\right)$ and, from Equation (4), we obtain n>−logα/(1−h(ν)) (for the most powerful test). It is known that the Shannon entropy is 1 if and only if ν is a uniform measure $\mu_{u}$. Therefore, in a certain sense, the difference 1−h(ν) estimates the distance between the distributions, and the last inequality shows that the sample size becomes infinite if ν approaches a uniform distribution.

The next simple example illustrates this theorem. Let there be a statistic τ and a generator (a measure ν) created sequences of binary digits which are independent and, say, ν(0)=0.501,ν(1)=0.499. Suppose, $\lim_{n\to \infty }-\frac{1}{n}$$\log\pi_{\tau }\left(x\right)$=c, where *c* is a positive constant. Let us consider the following “decimation test” $\tau^{1/2}$: an input sequence $x_{1}x_{2}\ldots .x_{n}$ is transformed into $x_{1}x_{3}x_{5}\ldots x_{2\lfloor n/2\rfloor -1}$ and then the test is applied to this transformed sequence. Obviously, for this test, $\lim_{n\to \infty }-\frac{1}{n/2}\log\pi_{\tau^{1/2}}\left(x\right)=c\;$ and, hence, $\lim_{n\to \infty }$$-\frac{1}{n}$$\log\pi_{\tau^{1/2}}\left(x\right)=c/2\;$. Thus, the value $-\frac{1}{n}\log\pi_{\tau }\left(x_{1}\ldots x_{n}\right)$ seems to be a reasonable estimate of the power of the test for a large *n*.

## 3. Time-Adaptive Testing and Their Experimental Investigation

### 3.1. Batteries of Tests

Let us consider a situation where the randomness testing is performed by conducting a battery of statistical tests for randomness. Suppose that the battery contains *s* tests and $\alpha_{i}$ is the significance level of *i*th test, i=1,…,s. If the battery is applied in such a way that the hypothesis $H_{0}$ is rejected when at least one test in the battery rejects it, then the significance level α of this battery satisfies the following inequality:

(6) $$\alpha \leq \sum_{i=1}^{s}\alpha_{i}\;.$$

If all the tests in the battery are independent, then the following equation is valid: $\alpha =1-\prod_{i=1}^{s}\left(1-\alpha_{i}\right)\;.$ Clearly, the upper bound in Equation (6) is true for this case and $1-\prod_{i=1}^{s}\left(1-\alpha_{i}\right)$ is close to $\sum_{i=1}^{s}\alpha_{i}$, if each $\alpha_{i}$ is much smaller than 1/s. That is why we use the estimate in Equation (6) below.

We considered a scenario in which a test is applied to a single sequence generated by an RNG, and then the researcher makes a decision on the RNG based on the test results. Another possibility that has been considered by several authors (e.g., [2,5]) is to use the following two-step procedure for testing RNGs. The idea is to generate *r* sequences $x^{1},x^{2},\ldots ,x^{r}$ and apply one test (say, τ) to each of them independently. Then, apply another test to the received data $\tau \left(x^{1}\right),\tau \left(x^{2}\right),\ldots ,\tau \left(x^{r}\right)$ (as a rule, those values are converted into a sequence of corresponding *p*-values, and then the hypothesis of the uniform distribution of those *p*-values is tested). Then, this procedure is repeated for the second test in the battery, and so on. The final decision is made on the basis of the results obtained. We do not consider this two-step procedure in detail, but note that time-adaptive testing can be applied in this situation, too.

### 3.2. The Scheme of the Time-Adaptive Testing

Let there be an RNG which generates binary sequences, and a battery of *s* tests with statistics $\tau_{1},\tau_{2},\ldots ,\tau_{s}$. In addition, suppose that the total available testing time is limited to a certain amount *T* and the level of significance is α∈(0,1).

When the time-adaptive testing is applied, all the calculations are separated into a preliminary stage and a final one. The result of the preliminary stage is the list of values

$$\gamma_{1}=\frac{-\log\pi_{\tau_{1}}\left(x_{1}^{1}x_{2}^{1}\ldots x_{n_{1}}^{1}\right)}{n_{1}},\gamma_{2}=\frac{-\log\pi_{\tau_{2}}\left(x_{1}^{2}x_{2}^{2}\ldots x_{n_{2}}^{2}\right)}{n_{2}}$$

(7) $$,\ldots ,\;\;\gamma_{s}=\frac{-\log\pi_{\tau_{s}}\left(x_{1}^{s}x_{2}^{s}\ldots x_{n_{s}}^{s}\right)}{n_{s}}\;.$$

Then, taking into account the values in Equation (7), it is possible to choose some tests from the battery and apply them to the longer sequence, calculate new values γ, and so on. When the preliminary stage is carried out, several tests from the battery should be chosen for the next stage.

The final stage is as follows. First, we divide the significance level α into $\alpha_{1},\alpha_{2},\ldots ,\alpha_{k}$ in such a way that $\sum_{i=1}^{k}\alpha_{i}=\alpha$. Then, we obtain new sequence(s) $y_{1}^{1}y_{2}^{1}\ldots y_{m_{1}}^{1},\ldots ,y_{1}^{k}y_{2}^{k}\ldots y_{m_{k}}^{k}$, which may have common parts, but are independent of $x_{1}^{1}x_{2}^{1}\ldots x_{n_{1}}^{1}$, $\ldots ,x_{1}^{s}x_{2}^{s}\ldots x_{n_{s}}^{s}$, and calculate

(8) $$\pi_{\tau_{i_{1}}}\left(y_{1}^{1}y_{2}^{1}\ldots y_{m_{1}}^{1}\right),\ldots ,\pi_{\tau_{i_{k}}}\left(y_{1}^{k}y_{2}^{k}\ldots y_{m_{k}}^{k}\right)\;.$$

The hypothesis $H_{0}$ is accepted, if $\pi_{\tau_{i_{j}}}\left(y_{1}^{j}y_{2}^{j}\ldots y_{m_{j}}^{j}\right)>\alpha_{j}$ for all j=1,…,k. Otherwise, $H_{0}$ is rejected. The parameters of the test should be chosen in such a way that the total time of calculation is not grater than the given limit *T*.

Note that, during a preliminary stage, the sequences $x_{1}^{1}x_{2}^{1}\ldots x_{n_{1}}^{1}$, $\ldots ,x_{1}^{s}x_{2}^{s}\ldots x_{n_{s}}^{s}$ may have common parts (for example, the first sequence may be the prefix of the second, etc.). The fact is that the final stage and the preliminary stage are statistically independent, and, therefore, the use of common parts is quite correct. On the other hand, this can affect the calculation time and, indirectly, the test result.

#### Claim 1

The significance level of the described time-adaptive test is not larger than α.

Indeed, the sequences $y_{1}^{1}y_{2}^{1}\ldots y_{m_{1}}^{1},\ldots ,y_{1}^{k}y_{2}^{k}\ldots y_{m_{k}}^{k}\;$ and $x_{1}^{1}x_{2}^{1}\ldots x_{n_{1}}^{1}$, $\ldots ,x_{1}^{s}x_{2}^{s}\ldots x_{n_{s}}^{s}$ are independent and, hence, the results of the final stage does not depend on the preliminary one. When the battery $\tau_{i_{1}},\tau_{i_{2}},\ldots ,\tau_{i_{k}}$ is applied, the significance level of $\tau_{i_{j}}$ equals $\alpha_{j}$ and the significance level of the battery equals $\sum_{i=1}^{k}\alpha_{i}$. From Equation (6), we can see that the significance level of the battery (and, hence, of the described testing) is not grater than α.

*Comment.* The length of the sequences may depend on the speed of tests. For example, it can be done as follows: let $v_{i}$ be the speed per bit of the test $\tau_{i}$, i=1,…,s. One possible way to take into account the speed difference is to calculate

$${\hat{\gamma }}_{i}=\frac{-\log\pi_{\tau_{i}}\left(x_{1}^{i}x_{2}^{i}\ldots x_{n_{i}}^{i}\right)}{n_{i}/v_{i}},\;\;\;i=1,\ldots ,s,$$

instead of Equation (7) and similar expressions.

### 3.3. The Experiments

We carried out some experiments which were intended to assess the potential ability of time-adaptive testing rather than to find the optimal design of the test.

For experiments, we took batteries Rabbit and Alphabit from [2], while RNGs were specially prepared. The point is that nowadays there are many “bad” PRNGs and “good” ones. In other words, the output sequences of some known PRNGs have some deviations from randomness, which are quite easy to detect with many known tests, while other PRNGs do not have deviations that can be detected by known tests [2]. Thus, we need to have some families of RNGs with such deviations from randomness that they can be detected only for quite large output sequences. To do this, we took a good generator MRG32k3a and a bad one LCG (with parameters *m* = 2,147,483,647, *a* = 16,807, *b* = 0, *c* = 12,345) from [2], generated sequences $g_{1}g_{2}\ldots$ and $b_{1}b_{2}\ldots$ by these two generators, and then prepared a “mixed” sequence $m_{1}m_{2}\ldots$ in such a way that

(9) $$m_{i}=\left\{\begin{array}{cc} g_{i} & \;\mathrm{if}\;i\;\mathrm{mod}\;D\;\;\neq 0\\ b_{i} & \;\mathrm{if}\;i\;\mathrm{mod}\;D\;\;=0 \end{array}\right.$$

where *D* is a parameter.

The time-adaptive testing was organized as follows: during the preliminary stage, we first generated a file $m_{1}m_{2}\ldots m_{l_{1}}$ with $l_{1}$ = 2,000,000 bytes, tested it by 25 tests from the Rabbit battery and calculated the values in Equation (7) with $\log\equiv \log_{2}$ (see the left part of Table 1). (This battery contains 26 tests, but one of them cannot be applied to such a short sequence.) Then, we chose five tests with the biggest value $-log\;\pi_{t_{i}}\left(m_{1}\ldots m_{l_{i}}\right)/l_{1}$ (let them be $t_{i_{1}},\ldots ,t_{i_{5}}$), generated a sequence $m_{1}\ldots m_{l_{2}}$ with $l_{2}$ = 6,000,000 bytes and applied the tests $t_{i_{1}},\ldots ,t_{i_{5}}$ for testing this sequence (see the example in the right part of Table 1). After that, we found a test $t_{f}$ for which

(10) $$-log\;\pi_{t_{f}}/l_{f}=\max_{r=1,\ldots ,25;\;j=i_{1}\ldots i_{5}}\left\{-log\pi_{r}\left(m_{1}\ldots m_{l_{1}}\right)/l_{1},-log\pi_{j}\left(m_{1}\ldots m_{l_{2}}\right)/l_{2}\right\}\;.$$

In other words, for $t_{f}$ the value $-log\pi_{r}\left(m_{1}\ldots m_{l_{k}}\right)/l_{k}$ is maximal for k=1,2 and all *r* (see the Table 1). The preliminary stage was finished. Then, during the second stage, we generated a 40,000,000 byte sequence, and applied the test $t_{f}$ to it. If the obtained *p*-value was less than 0.001, the hypothesis $H_{0}$ was rejected. (Note that the sequence length $l_{1}$ = 2,000,000 and $l_{2}$ = 6,000,000 are 5% and 15% from the final length of 40,000,000 bytes. Thus, the total length of the sequences tested by all the tests during the preliminary stage is 25×0.05+5×0.15=2 the final length, i.e., 2 × 40,000,000. If we take into account the second stage, the total length is 3 × 40,000,000. On the other hand, if one applies the battery Rabbit to the sequence of the same length, the total length of investigated sequences is 25 × 40,000,000, i.e., 8.33 times more.

Let us consider one example in detail, taking D=2 in Equation (9).

Table 1 contains the calculation results where 25 tests from the Rabbit battery were applied to a sequence of 2,000,000 bytes. Then, five tests with the smallest *p* values were applied to the sequence of 6,000,000 bytes (see Table 2). After that, we calculated Equation (10) and found the that the value $-\log_{2}\pi /l$ is maximal for the test t13. The preliminary stage was finished. Then, at the final stage, we applied the test t13 to the new 40,000,000-byte sequence. It turned out that $\pi_{t13}=$$2.9\times 10^{-26}$ and, hence, $H_{0}$ is rejected. Besides, we estimated time of all calculation (during both stages).

After that, we conducted an additional experiment to get the full picture. Namely, we calculated *p*-values for all tests for the same 40,000,000-byte sequence and then estimated the total time of calculations. It turned out that the *p*-values of the two tests were less than 0.001. Namely, $\pi_{t13}=$$2.9\times 10^{-26}$ and $\pi_{t22}=$$1.1\times 10^{-6}$. (Note that *p*-value of this test is 0.12 for the 6,000,000-byte file.) Besides, we estimated time of calculations for all experiments. Thus, the described time-adaptive testing revealed one of the two most powerful tests, while the time used is eight times.

Table 3 describes an experiment with the battery Alphabet. The parameter *D* in Equation (9) was 4 and the length of a sequence was 60,000,000 bytes. During the preliminary stage, the 3,000,000 sequence was generated and tested by all tests. Then, four tests with the smallest *p*-values were applied to the sequence of 9,000,000 bytes and then we calculated Equation (10) and found the that the value $-\log_{2}\pi /l$ is maximal for the test t15. After that, this test was applied to 60,000,000-byte sequence. As in the previous example, we calculated the *p*-values for all tests on the same 60,000,000-byte sequence. All *p*-values are presented in Table 3.

We carried out similar experiments for different D=2,3,4 (in Equation (9)) with different good and bad generators from [2] for batteries Rabbit and Alphabet. It turned out that in all cases either the battery rejects $H_{0}$ and the time-adaptive testing also rejects $H_{0}$ or $H_{0}$ was not rejected by both tests.

## 4. Conclusions

In this article, we show that the proposed time-adaptive testing is promising for RNG testing. On the other hand, the proposed time-adaptive testing does not offer exact values of numerous parameters for all possible batteries. Among these parameters, we note the number of steps at the preliminary stage (in the considered example, there were two such steps: selecting five tests and then one), the number of tests compared in one step, the length of the tested sequences, the rule for choosing tests at different stages, etc. The problem of parameter selection can be considered a multidimensional optimization problem. There are many methods available for solving such problems (for example, neural networks and other AI algorithms), and some of them can be used along with the time-adaptive testing.

We believe that the proposed approach makes it possible to investigate and optimize time-adaptive testing.

## Figures and Tables

**Table 1:** Time-adaptive testing. Preliminary stage.

Test	Length (*l*) (Bytes)	*P*-Value (π)	−log${}_{2}\pi /l$
t1	2 $\times 10^{6}$	0.42	$6.3\times 10^{-7}$
t2	$2\times 10^{6}$	0.37	$7.2\times 10^{-7}$
t3	$2\times 10^{6}$	0.028	$26\times 10^{-7}$
t4	$2\times 10^{6}$	0.78	$1.8\times 10^{-7}$
t5	$2\times 10^{6}$	0.4	$6.6\times 10^{-7}$
t6	$2\times 10^{6}$	0.37	$7.2\times 10^{-7}$
t7	$2\times 10^{6}$	0.059	$20\times 10^{-7}$
t8	$2\times 10^{6}$	0.026	$26\times 10^{-7}$
t9	$2\times 10^{6}$	0.72	$2.4\times 10^{-7}$
t10	$2\times 10^{6}$	0.72	$2.4\times 10^{-7}$
t11	$2\times 10^{6}$	0.63	$3.3\times 10^{-7}$
t12	$2\times 10^{6}$	0.74	$2.2\times 10^{-7}$
t13	$2\times 10^{6}$	0.021	$28\times 10^{-7}$
t14	$2\times 10^{6}$	0.42	$6.2\times 10^{-7}$
t15	$2\times 10^{6}$	0.9	$0.76\times 10^{-7}$
t16	$2\times 10^{6}$	0.087	$18\times 10^{-7}$
t17	$2\times 10^{6}$	0.72	$2.3\times 10^{-7}$
t18	$2\times 10^{6}$	0.58	$3.9\times 10^{-7}$
t19	$2\times 10^{6}$	0.89	$0.84\times 10^{-7}$
t20	$2\times 10^{6}$	0.51	$4.9\times 10^{-7}$
t21	$2\times 10^{6}$	0.047	$22\times 10^{-7}$
t22	$2\times 10^{6}$	0.47	$0.47\times 10^{-7}$
t23	$2\times 10^{6}$	0.18	$12\times 10^{-7}$
t24	$2\times 10^{6}$	0.14	$14\times 10^{-7}$
t25	$2\times 10^{6}$	0.024	$27\times 10^{-7}$

**Table 2:** Time-adaptive testing. Preliminary stage.

Test	Length (*l*) (Bytes)	*P*-Value	−log${}_{2}\pi /l$
t3	6 $\times 10^{6}$	0.23	$3.5\times 10^{-7}$
t8	$6\times 10^{6}$	0.0037	$13\times 10^{-7}$
t13	$6\times 10^{6}$	0.0028	$14\times 10^{-7}$
t21	$6\times 10^{6}$	0.73	$0.76\times 10^{-7}$
t25	$6\times 10^{6}$	0.05	$7.2\times 10^{-7}$

**Table 3:** *p*-values for different tests from Alphabet.

Test	Step 1	Step 2	Step 3	Alphabet
1	0.4	-	-	0.37
2	0.27	-	-	0.47
3	0.28	-	-	0.047
4	0.056	0.057	-	$2.9\times 10^{-7}$
5	0.064	-	-	0.021
5	0.38	-	-	0.0027
6	0.28	-	-	0.22
7	0.21	-	-	0.18
8	0.36	-	-	0.29
9	0.31	-	-	0.16
10	0.091	-	-	0.15
11	0.032	0.087	-	0.33
12	0.12	-	-	0.032
13	0.19	-	-	0.19
14	0.055	0.073	-	0.12
15	0.045	0.042	3.6$\times 10^{-6}$	$3.6\times 10^{-6}$
16	0.091	-	-	0.26
17	0.24	-	-	0.31

## Appendix A. Consistent Tests Based on Universal Codes

The considered tests are based on so-called universal codes, which is why we first briefly describe them. For any integer *m*, a code ϕ is defined as such a map from the set of *m*-letter words to the set of all binary words that for any *m*-letter *u* and *v*ϕ(u)≠ϕ(u). This property gives a possibility to uniquely decode. (More formally, ϕ is injective mapping from ${\left\{0,1\right\}}^{m}$ to ${\left\{0,1\right\}}^{*}$, where ${\left\{0,1\right\}}^{*}={⋃}_{i=1}^{\infty }{\left\{0,1\right\}}^{i}$.) We consider so-called universal codes which have the two following properties:

(A1) $$\forall \;m>0\;\sum_{u\in {\left\{0,1\right\}}^{m}}2^{-|\phi (u)|}=1\;$$

and for any stationary ergodic ν defined on the set of all infinite binary words $x=x_{1}x_{2}\ldots$, with probability one

(A2) $$\lim_{n\to \infty }\frac{1}{n}\left|\phi \left(x_{1}x_{2}\ldots x_{n}\right)\right|/n=h\left(\nu \right)\;$$

where h(ν) is the Shannon entropy of ν. Such code exists (see [7]). Note that a goal of codes is to “compress” sequences, i.e., make an average length of the codeword $\phi (x_{1}x_{2}\ldots x_{n})$ as small as possible. The second property in Equation (A2) shows that the universal codes are asymptotically optimal, because the Shannon entropy is a low bound of the length of the compressed sequence (per letter) (see [7]).

Let us return to the considered problem of hypothesis testing. Suppose it is known that a sample sequence $x=x_{1}x_{2}\ldots$ was generated by stationary ergodic source and, as before, we consider the same $H_{0}$ against the same $H_{1}$. Let ϕ be a universal code. The following test is suggested in [13]:

If the length $\left|\phi (x_{1}\ldots x_{n}\right)$$\leq n-\log_{2}\alpha$, then $H_{0}$ is rejected, otherwise accepted. Here, as above, α is the significance level and $\left|\phi (x_{1}\ldots x_{n})\right|$ is the length of encoded (“compressed”) sequence. We denote this test by $T_{\phi }$ and its statistic by $\tau_{\phi }$, i.e.,

(A3) $$\tau_{\phi }\left(x_{1}\ldots x_{n}\right)=n-\left|\phi \left(x_{1}\ldots x_{n}\right)\right|\;.$$

The following theorem is proven in [13,14]:

**Theorem** **A1.**
*For each stationary ergodic ν, alpha∈(0,1), and a universal code ϕ, with probability 1, the Type I error of the described test is not larger than α and the Type II error goes to 0, when n→∞.*

## Appendix B. Proofs

**Proof of Theorem 1.** The known Shannon–McMillan–Breiman (SMB) theorem claims that, for the stationary ergodic source ν and any ϵ>0,δ>0, there exists such $n^{\prime }$ that

(A4) $$\nu \{x:x\in {\left\{0,1\right\}}^{n}\;\;\;\&\;\;\;h\left(\nu \right)-\epsilon <-\frac{1}{n}\log\nu \left(x\right)<h\left(\nu \right)+\epsilon \;\;\}>1-\delta \;$$

for $n>n^{\prime }$ (see [7]). From this, we obtain

(A5) $$\nu \{x:x\in {\left\{0,1\right\}}^{n}\;\;\;\&\;\;2^{-n(h(\nu )-\epsilon )}>\;\nu \left(x\right)>2^{-n(h(\nu )+\epsilon )}\}>1-\delta \;$$

for $n>n^{\prime }$. It is convenient to define

(A6) $$\Phi_{\epsilon ,\delta ,n}=\left\{x:x\in {\left\{0,1\right\}}^{n}\;\;\;\&\;\;\;h\left(\nu \right)-\epsilon <-\frac{1}{n}\log\nu \left(x\right)<h\left(\nu \right)+\epsilon \;\;\right\}$$

From this definition and Equation (A5), we obtain

(A7) $$\left(1-\delta \right)\;2^{n(h(\nu )-\epsilon )}\leq \left|\Phi_{\epsilon ,\delta ,n}\right|\leq 2^{n(h(\nu )+\epsilon )}\;.$$

For any $x\in \Phi_{\epsilon ,\delta ,n}$, define

(A8) $$\Lambda_{x}=\left\{y:\nu \left(y\right)>\nu \left(x\right)\;\;\right\}⋂\Phi_{\epsilon ,\delta ,n}\;.$$

Note that, by definition, $|\Lambda_{x}\left|\leq \right|\Phi_{\epsilon ,\delta ,n}|$ and, from Equation (A7), we obtain

(A9) $$|\Lambda_{x}|\leq 2^{n(h(\nu )+\epsilon )}\;.$$

For any ρ∈(0,1), we define $\Psi_{\rho }\subset \Phi_{\epsilon ,\delta ,n}$ such that

(A10) $$\nu \left(\Psi_{\rho }\right)=\rho \;\;\&\;\;\forall u\in \Psi_{\rho }\;\forall v\in \left(\Phi_{\epsilon ,\delta ,n}\setminus \Psi_{\rho }\right)\;\to \nu \left(u\right))\geq \nu \left(v\right)\;.$$

(That is, $\Psi_{\rho }$ contains the most probable words whose total probability equals ρ.) Let us consider any $x\in (\Phi_{\epsilon ,\delta ,n}\setminus \Psi_{\rho })$. Taking into account the definition in Equations (A10) and (A7), we can see that for this *x*

(A11) $$|\Lambda_{x}\left|\geq \rho \right|\Phi_{\epsilon ,\delta ,n}|\geq \rho \left(1-\delta \right)2^{n(h(\nu )-\epsilon )}\;.$$

Thus, from this inequality and Equation (A9), we obtain

(A12) $$\rho \left(1-\delta \right)2^{n(h(\nu )-\epsilon )}\leq \left|\Lambda_{x}\right|\leq \;2^{n(h(\nu )+\epsilon )}\;.$$

From Equations (A5), (A6), and (A10), we can see that $\nu \left(\Phi_{\epsilon ,\delta ,n}\setminus \Psi_{\rho }\right)\geq \left(1-\delta \right)\left(1-\rho \right)$. Taking into account Equation (A12) and this inequality, we can see that

$$\nu \{x:x\in {\left\{0,1\right\}}^{n}\&h\left(\nu \right)-\epsilon -\log\left(\rho \left(1-\delta \right)\right)/n\leq \log|\Lambda_{x}|/n$$

(A13) ≤h(ν)+ϵ}≥(1−δ)(1−ρ).

From the definition in Equation (2) of $\pi_{NP}\left(x\right)$ and the definition in Equation (A8) of $\Lambda_{x}$, we can see that $\pi_{NP}\left(x\right)=\left|\Lambda_{x}\right|/2^{n}$. Taking into account this equation and Equation (A13), we obtain the following:

$$\nu \{x:x\in {\left\{0,1\right\}}^{n}\;\&\;1-\left(h\left(\nu \right)-\epsilon -\log\left(\rho \left(1-\delta \right)\right)/n\right)\geq$$

(A14) $$-\log\pi_{NP}\left(x\right)/n\geq 1-\left(h\left(\nu \right)+\epsilon \right)\}\geq \left(1-\delta \right)\left(1-\rho \right).$$

Having taken into account that this inequality is valid for all positive ϵ,δ, and ρ, we obtain the first statement of the theorem. The proof of the second statement of the theorem is close to the previous one. First, from Theorem A1 we see that, for any ϵ>0,δ>0, we define

(A15) $${\hat{\Phi }}_{\epsilon ,\delta ,n}=\{x:h\left(\nu \right)-\epsilon <|\phi \left(x_{1}\ldots x_{n}\right)\left|/n<h\left(\nu \right)+\epsilon \;\right\}\;.$$

Note that, from Equation (A2), we can see that there exists such $n^{\prime \prime }$ that, for $n>n^{\prime \prime }$,

(A16) $$\nu ({\hat{\Phi }}_{\epsilon ,\delta ,n})>1-\delta \;.$$

We use the set $\Phi_{\epsilon ,\delta ,n}$ (see Equation (A6)). Having taken into account the SMB theorem in Equations (A4) and (A16), we can see that

(A17) $$\nu ({\hat{\Phi }}_{\epsilon ,\delta ,n}\cap \Phi_{\epsilon ,\delta ,n})>1-2\delta \;,$$

if $n>\max(n^{\prime },n^{\prime \prime })$. From this moment, the proof begins to repeat the proof of the first statement if we use the set $\left({\hat{\Phi }}_{\epsilon ,\delta ,n}\cap \Phi_{\epsilon ,\delta ,n}\right)$ instead of $\Phi_{\epsilon ,\delta ,n}$. The only difference is in the definitions in Equations (A8) and (A10) which should be changed as follows.

$$\Lambda_{x}=\left\{y:|\phi |\left(y\right)|<|\phi \left(x\right)|\;\;\right\}\cap \left({\hat{\Phi }}_{\epsilon ,\delta ,n}\cap \Phi_{\epsilon ,\delta ,n}\right)\;$$

and $\Psi_{\rho }$ is such a subset of $\left({\hat{\Phi }}_{\epsilon ,\delta ,n}\cap \Phi_{\epsilon ,\delta ,n}\right)$ that

$$\nu \left(\Psi_{\rho }\right)=\rho \;\;\&\;\;\forall u\in \Psi_{\rho }\;\forall v\in \left(\Phi_{\epsilon ,\delta ,n}\setminus \Psi_{\rho }\right)\;\to |\phi \left(u\right)|\leq |\phi \left(v\right)|\;.$$

If we replace $\pi_{NP}$ with $\pi_{\tau_{\phi }}$ and δ with 2δ, we obtain the proof of the second statement. The theorem is proven. □
